# Study on the Influence of Weld Spacing on the Tensile Strength of Laser Double-Pass Reciprocating Welding of DP780/6061-T6 Dissimilar Metals

**DOI:** 10.3390/ma16072560

**Published:** 2023-03-23

**Authors:** Yaowu Zhao, Xueqian Qin, Yuhong Long, Jia Zhou, Hui Jiao

**Affiliations:** Guangxi Key Laboratory of Manufacturing Systems and Advanced Manufacturing Technology, School of Mechanical & Electrical Engineering, Guilin University of Electronic Technology, Guilin 541004, Chinajh1020116567@163.com (H.J.)

**Keywords:** laser welding, steel aluminum dissimilar metal, weld spacing, preheating, temperature field

## Abstract

The welding of steel–aluminum dissimilar metals plays a vital role in promoting automobile lightweight. However, it is tricky to obtain good mechanical properties of steel–aluminum laser weldments. Based on the principle of preheating welding, the laser double-pass reciprocating welding method of steel–aluminum dissimilar metals was proposed. In the experiment, different weld spacing such as 0, 0.5, 1.0, 1.5, and 2.0 mm were set, and numerical calculations of the temperature field of the molten pool were carried out. The results show that the tensile strength of weldment depends on the mechanical properties of the second weld seam in the optimal welding parameters. Compared with other weld spacing, when the weld spacing is 1.5 mm, the preheating temperature, peak temperature, and pool width on the steel side of the second weld are lower. In contrast, the weld penetration’s peak value and molten pool center’s temperature reach the maximum on the aluminum side. The thickness of the steel/aluminum transition layer changed from 14 to 11 to 8 μm with increased weld spacing. Moreover, the fracture mode of the second weld is a ductile fracture. Furthermore, the average tensile strength can reach 76.84 MPa. The results show that appropriate weld spacing and preheating temperature can effectively improve the tensile strength of the welding joint.

## 1. Introduction

The welding of dissimilar steel–aluminum metals helps develop the characteristics of different metals and has important application value in lightweight automobiles [1]. However, brittle intermetallic compounds (IMCs) are inevitably formed in the steel aluminum dissimilar metals’ welding, which deteriorates the mechanical properties of the weldment. Various welding methods have been applied to the connection of steel–aluminum dissimilar metals such as friction stir welding [2,3,4,5], cold metal transfer (CMT) welding [6,7,8], resistance spot welding [9,10,11], and laser welding [12,13,14]. Among them, friction stir welding requires special fixtures only suitable for flat plate welding. CMT welding equipment is expensive. Resistance welding requires space to be held at the upper and lower ends of the welded part. Laser welding is considered as a desirable choice for dissimilar metals welding due to its non-contact nature, high energy density, precise control over the heat input, and convenience of automation [15]. However, laser welding of steel-aluminum dissimilar metals still has problems, such as the formation of IMCs.

Relevant studies have shown that preheating can reduce the heat input, temperature difference, and residual stress during welding, and affect the thickness of the IMCs, the tensile strength of the welded joint, and the characteristics of the fracture surface of the weld [16,17,18]. At present, the relevant experiments are mainly realized by the overall preheating of the plate, the local preheating by double laser beams, and the local preheating by hybrid welding.

Hong Ma et al. [19] studied the microstructure and mechanical properties of brazed-fusion welded joint of aluminum alloy to galvanized steel at different preheating temperatures. The results show that preheating can improve the spreadability of the weld seam and change the thickness and phase composition of the IMCs layer. When the preheating temperature is 100 K, the tensile strength of the welded joint reaches the maximum, which is close to 80% of the aluminum alloy base metal. The fracture surface of the welded joint is located in the heat-affected zone of the aluminum alloy.

Ti-22Al-25Nb and TA15 alloys were welded using continuous dual-beam laser welding [20]. The first laser spot preheats the weld. The research shows that when the laser power ratio increases from 30:70 to 40:60, the tensile strength increases from 1016 Mpa to 1206 Mpa. However, when the laser spot spacing is 0.54 mm or the power ratio is 50:50, the laser cannot penetrate the material.

Dissimilar metal welding of Q235 low carbon steel and 5052 aluminum alloy was carried out by a single/dual-beam laser by Shuhai Chen et al. [21] The results showed that dual-beam laser welding, compared with single-beam laser welding, had better process stability, which made for a better weld appearance and a bigger effective joining width, enhancing tensile capacity.

Hongbo Xia et al. [22] used different laser beam modes (single beam, cross beam, and straight double beam) to braze DP590 and 6061-T6 dissimilar metals. The results show that when crossed dual beams are used, the welds are well formed, the IMCs are most uniform, and joints with the highest tensile strength are produced.

Masoud Mohammadpour et al. [23] used a dual laser beam-brazing process to join two types of galvanized steel and Al6022 aluminum alloy. The results show that the double-beam laser shape and high scanning speed can control the thickness of IMCs to 3 μm, and change the failure position from the steel brazing interface to the aluminum brazing interface.

By adjusting the thermal history of the laser welding process, the microstructure of the welded joint can be adjusted to improve the welding performance.

However, using a heating plate to preheat the whole base metal is less efficient. Using double beams for preheating base metal requires higher requirements on the beam transmission system of the laser, which makes the laser processing equipment expensive. The composite welding process is complicated. In addition, the preheating temperature of laser welding steel-aluminum dissimilar metals needs more in-depth research papers to improve the welding quality. In order to study the effect of preheating temperature on the welding of steel and aluminum dissimilar metals and simplify the preheating process, a single laser beam is used for double-pass reciprocating welding. After the first weld is welded, the residual temperature on the substrate is used as the preheating temperature of the second weld. The preheating temperature of the second weld is changed by adjusting the weld spacing. The influence of weld spacing and preheating temperature on the second weld pool’s morphology and the welded joint’s overall mechanical properties were studied. Compared with traditional preheating and double-beam welding, laser double-pass reciprocating welding has the characteristics of a simple process and convenient operation.

## 2. Experimental Procedure

### 2.1. Welding Trials

**Figure 1:**
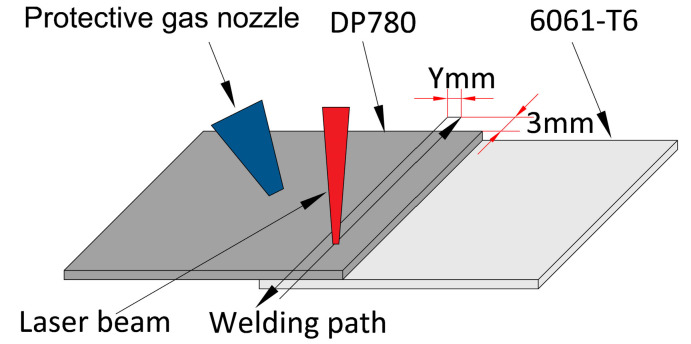
Schematic diagram of laser double-pass reciprocating welding.

6061-T6 aluminum alloy and DP780 steel, widely used in industrial manufacturing, were selected as experimental materials. The chemical composition of steel and aluminum alloy base metal is listed in Table 1. The steel and aluminum base metal size is 30 mm × 30 mm × 1 mm. Before welding, the surface of the parts to be welded of base metal is polished with 400 mesh and 800 mesh sandpaper to remove the oxide layer, then cleaned with water. An IPG fiber laser of 480 W in power was used to carry out the welding. The steel plate is above the aluminum plate, and Ar gas is used as a protective gas, as shown in Figure 1. In the laser double-pass reciprocating welding, the weld spacing Y values between the two welds were set as 0, 0.5, 1.0, 1.5, and 2.0 mm. Six samples were welded with different laser parameters in each group. The samples with the maximum and minimum tensile strength values are discarded. The remaining four samples were adopted.

In the laser welding process, the parameters of the first and second weld remain unchanged. The universal electronic tensile test machine (WDW-10) was used to measure the tensile property of the welded joint. The Fe-Al transition layer is not parallel to the tensile direction in the tensile experiments for weldment of steel–aluminum deep laser penetration welding. Therefore, the Fe-Al transition layer is not solely subject to shear stress. Many papers refer to the tensile experiment as “tensile strength test” or “tensile shear test” and use “Mpa” or “N/mm” as the unit of measurement to describe the test results [24,25]. They are referring to the same test process. This paper uses “tensile strength” to describe the tensile test result.

The metallographic specimens of the weldments cross-section were prepared by discharge-cutting, mechanical grounding, and polishing. The microstructures were analyzed by optical microscopy (OM, OLYMPUS-OLS4100, OLYMPUS, Tokyo, Japan) and scanning by electron microscopy (SEM, Quanta FEG450, State of Orego, US) with an energy dispersive spectroscopy (EDS, X-Max20, Oxford Instruments, Oxford, UK).

### 2.2. Numerical Model of Temperature Field

**Figure 2:**
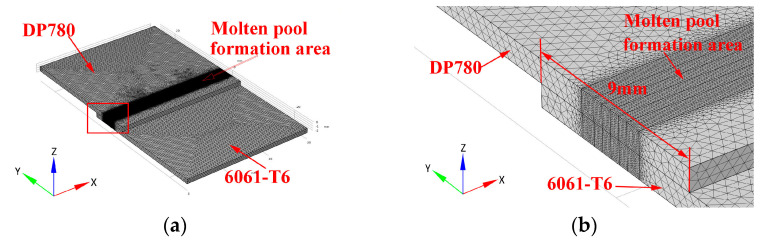
The appearance of the object mesh in the numerical model. (**a**) Overall view; (**b**) partially enlarged view of the red frame area in Figure 2a.

The numerical model of the temperature field in laser welding of steel and aluminum based on a simplified body heat source was established by COMSOL6.0 software. In the numerical model, only the solid heat conduction process is considered, without considering the phase transition between solid and liquid and the fluid movement in the molten pool. Additionally, the solid area where the temperature exceeds the melting point of the based metal is considered to be the molten pool area. The size of the based metal parts in the model is the same as the real ones. The lap width of weldment is 9 mm, as shown in Figure 2. In the weld seam area, the model is divided by a 0.1 mm free tetrahedral mesh, and the time-step was 0.003 s. The ambient temperature in the model is 300 K.

The simplified body heat source in the numerical model consists of three parts:

- Gauss heat source on the upper surface of the steel body (z=0 mm );
- Cylindrical heat source inside the steel body (−1 mm<z<0 mm);
- Semi-elliptical heat source inside the aluminum body (z≤−1 mm);
- The heat source model formula is as follows:

$$Q(x,y,z)\left\{ \begin{array}{l} \frac{\eta P}{R_{0}^{2}}\exp(\frac{-2({\left(x-x_{1}\right)}^{2}+{\left(y-y_{1}\right)}^{2})}{R_{0}^{2}})\;z=0\;\mathrm{mm}\\ (\eta_{1}\frac{z}{H}+\eta_{2})•\frac{P}{R_{1}^{2}}•\exp(\frac{-2({\left(x-x_{1}\right)}^{2}+{\left(y-y_{1}\right)}^{2})}{R_{1}})\;-1\;\mathrm{mm}<z<0\;\mathrm{mm}\\ \frac{2P}{\left(\pi x_{2}y_{2}z_{2}\right)}(\eta_{2}(x-x_{1}{)}^{2}+\eta_{3}{\left(y-y_{1}\right)}^{2}+\eta_{4}{\left(z-z_{1}\right)}^{2})<R_{2}^{2}\;z\leq -1\;\mathrm{mm} \end{array}\right.$$

where Q(x,y,z) is the heat flux; η is the absorption rate of the base metal to the laser; P is the laser power; $x_{1}$ is the x-coordinate of the laser spot center; $y_{1}$ is the y-coordinate of the laser spot center; $z_{1}$ is the z-coordinate of the laser spot center; $R_{0}$ is the reference value of the molten pool radius on the steel surface; $\eta_{1}$ and $\eta_{2}$ are the empirical parameters; $R_{1}$ is the reference value of the radius of the molten pool inside the steel body; $x_{2}$ and $y_{2}$ are the empirical value of the molten pool radius on the aluminum body; $z_{2}$ is the empirical value of the weld penetration on the aluminum side; $R_{2}$ is the reference value of the radius of the molten pool inside the aluminum body.

## 3. Results and Discussion

### 3.1. Tensile Strength of the Weldment

**Figure 3:**
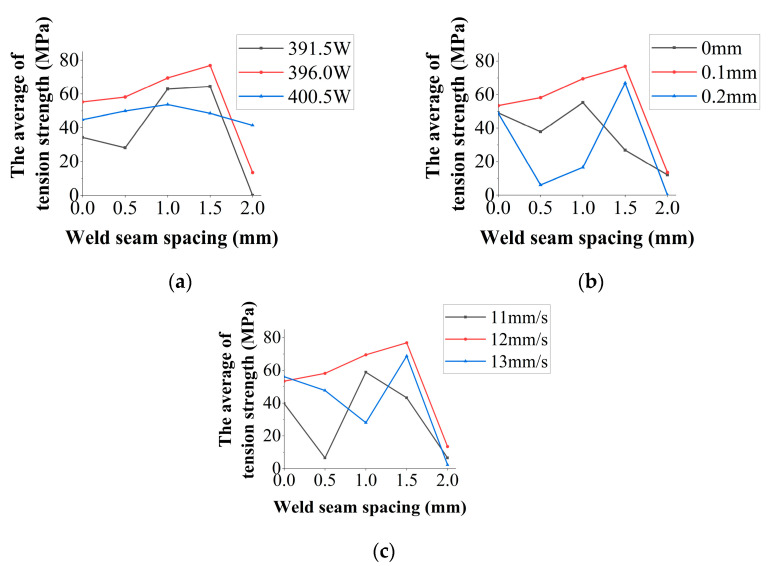
The suitable laser welding parameters are based on a single variable principle. (**a**) Change laser power; (**b**) change weld speed; (**c**) change defocusing distance.

In research of suitable laser welding parameters, the single laser power change is 4.5 W. The single change of defocusing distance was 0.1 mm. The single change of welding speed is 1 mm/s. The shielding gas flow rate is constant at 15 L/min. The optimal laser welding process with different weld spacing was tested according to the single variable principle. The results show that when the weld spacing is 0–1.5 mm, the optimal laser welding parameters are the same: laser power 396 W, defocus 0.1 mm, and welding speed 12 mm/s, as shown in Figure 3.

In Figure 3, when the weld spacing is 0–1.5 mm, the tensile strength of the weldments also increases with the weld spacing, up to 76.84 MPa. However, when the weld spacing increased to 2 mm, the tensile value of the welded joint decreased to 13.47 MPa. Hence, the tensile value provided by the first weld seam for the overall welded joint does not exceed 13.47 MPa.

When the second weld is welded, the molten pool of the first weld solidifies. The weld spacing does not affect the morphology and mechanical properties of the first weld. By comparing the tensile strength of the weldment with weld spacing of 1.5 mm and 2.0 mm, it can be seen that the second weld seam mainly determines the tensile strength of the whole weldment.

### 3.2. Analysis of Weld Cross-Section Appearance

**Figure 4:**
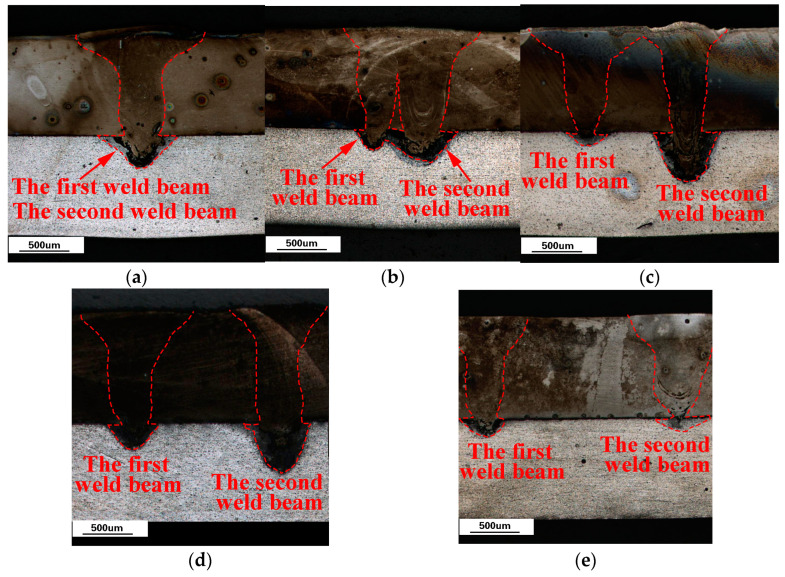
The cross-sectional appearance of the weldment with different weld spacing. (**a**) 0 mm weld spacing; (**b**) 0.5 mm weld spacing; (**c**) 1.0 mm weld spacing; (**d**) 1.5 mm weld spacing; (**e**) 2.0 mm weld spacing.

With the optimal laser welding parameters, the cross-sectional appearance of the weldment with different weld spacing is shown in Figure 4.

**Figure 5:**
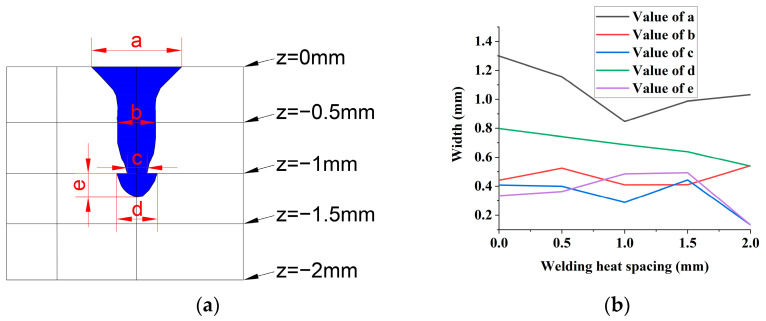
The size of the molten pool at the cross-section of the second weld seam at different weld spacing. (**a**) Measurement location of the molten pool; (**b**) dimension of the molten pool.

In Figure 4, when the weld spacing is 0 mm, the path of the first welding seam of the laser coincides with the second welding seam. When the welding spacing is 0.5 mm, the molten pool of the first weld overlaps partially with that of the second weld. When the weld spacing is 1.0 mm, 1.5 mm, and 2.0 mm, the molten pool morphology of the first weld seam is the same. However, the molten pool size of the second weld seam showed a different trend, as shown in Figure 5.

Compared with the second weld size of other weld spacing, when the weld spacing is 1.5 mm, the pool width “a” at the top of the steel side, “b” at the middle of the steel side, and “d” at the top of the aluminum side are all more minor. However, the pool width “c” at the bottom of the steel side and the weld penetration “e” of the aluminum side reached the maximum value.

Therefore, the morphological characteristics of the second weld seam that can provide the highest tensile strength for the whole weldment are the smaller pool width of the weld at the top and middle of the steel side and the largest weld penetration of the weld at the aluminum side. This means that the plasma shielding and smoke-blocking effects are minor during the second weld. Additionally, more laser energy reached the bottom of the laser keyhole to increase the weld penetration of the aluminum side.

### 3.3. Numerical Simulation Results of Temperature Field

**Figure 6:**
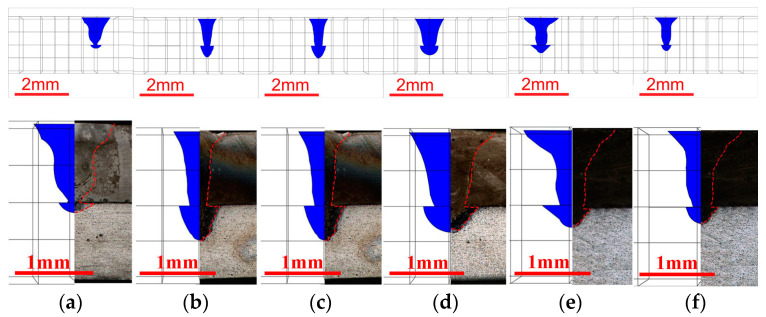
Comparison of simulated molten pool morphology and actual one. (**a**) The first weld seam; (**b**) the second weld seam with weld spacing of 0 mm; (**c**) the second weld seam with weld spacing of 0.5 mm; (**d**) the second weld seam with weld spacing of 1.0 mm; (**e**) the second weld seam with weld spacing of 1.5 mm; (**f**) the second weld seam with weld spacing of 2.0 mm.

The COMSOL software simulated the weld pool’s shape on the weldment’s cross-section. The simulated molten pool morphology was compared with the actual one, as shown in Figure 6.

**Figure 7:**
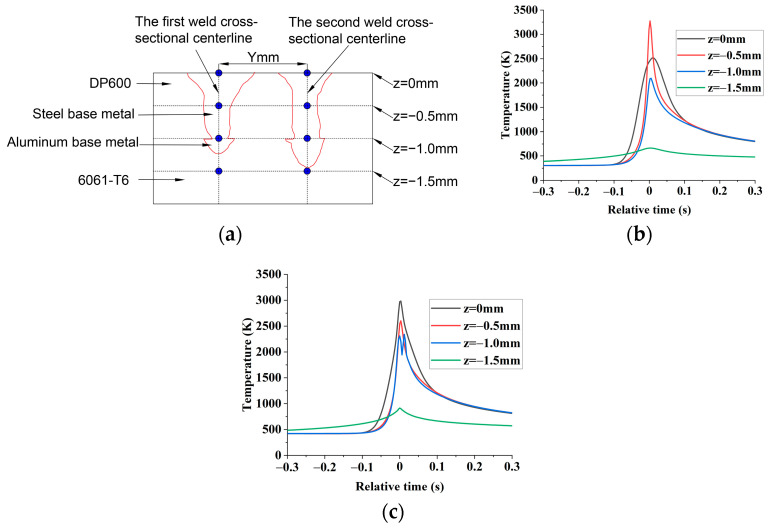
Time−temperature history of monitoring points from weldment cross-section. (**a**) Location of temperature monitoring points of weld cross-section; (**b**) time-temperature diagram of monitoring points of the first weld; (**c**) time-temperature diagram of the monitoring point of the second weld with weld spacing of 1.5 mm.

In Figure 6, the shape of the molten pool obtained by simulation is consistent with that of the actual molten pool. Therefore, the numerical simulation results of the temperature field are reliable. The monitoring point is taken from the center of the weld cross-section, as shown by the blue points in Figure 7a. The time the laser spot center passes through the section along the weld direction is taken as relative zero time. Moreover, extract the time-temperature history of the monitoring points, as shown in Figure 7b,c.

In Figure 7b,c, when −0.3  s<t<−0.25  s, the temperature of monitoring points of the same weld changes slowly. Therefore, the temperature at t=−0.3 s can be regarded as the preheating temperature of the second weld seam.

The thermal conductivity of aluminum and steel are 238 [W/(m × k)] and 22 [W/(m × k)], respectively. The thermal conductivity of aluminum is 10.8 times that of steel. Therefore, compared with the steel base metal, the aluminum base metal of the second weld is more susceptible to the temperature field of the first weld. Moreover, when the pool width and penetration depth of the molten pool on the aluminum side at the front end of the second welding seam is relatively large, the initial welding temperature at the rear end of the second welding seam will also be increased. Therefore, when the weld spacing is 1.5 mm, the preheating temperature of the second weld is 486 K, reaching the maximum. The aluminum side weld penetration of the second weld also reached the maximum.

**Figure 8:**
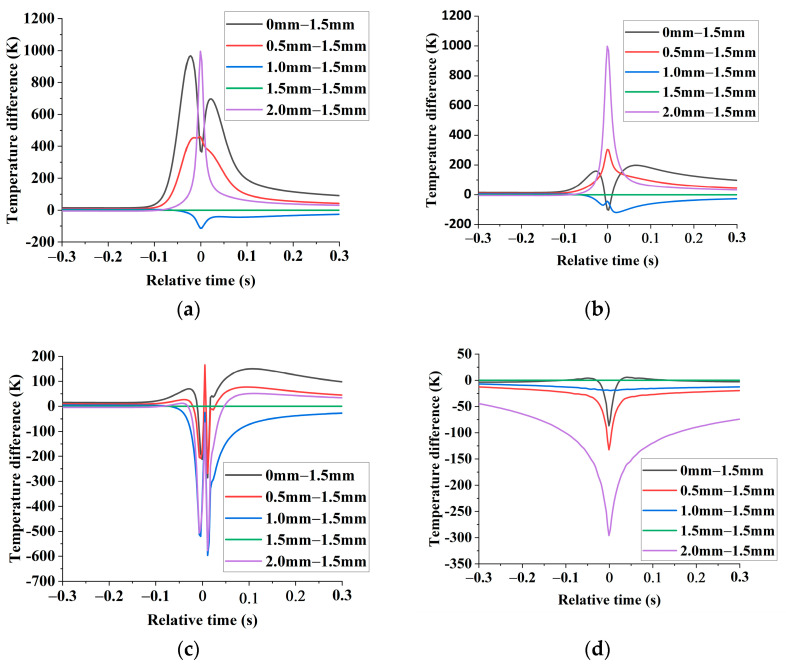
Compared with 1.5 mm weld spacing, the difference between the monitoring points of the same z value of the second weld at other weld spacing. (**a**) monitoring points (z=0 mm); (**b**) monitoring points (z=−0.5 mm); (**c**) monitoring points (z=−1.0 mm); (**d**) monitoring points (z=−1.5 mm).

Compared with 1.5 mm weld spacing, the temperature difference values of monitoring points of the second weld with different weld spacing is shown in Figure 8.

In Figure 8, compared with other weld spacing, when the weld spacing is 1.5 mm, the temperature peak value at z=0 mm is the lowest, the temperature peak at z=−0.5 mm is lower, and the temperature peaks at z=−1.0 mm and z=−1.5 mm are the highest. Therefore, when the weld spacing is 1.5 mm, in the second pass, more laser energy is concentrated at the bottom of the keyhole, closer to the aluminum base metal. On the other hand, the upper surface of the steel and the steel base metal area obtain less laser energy, resulting in a shallow pool width at the top and middle of the steel side. In contrast, the aluminum side has the most profound weld penetration.

When the weld spacing is 0–1.5 mm, with the increase in the weld spacing, the steel side preheating temperature of the second welding seam decreases, and the plasma shielding effect and the smoke blocking effect on the laser are reduced. The laser energy is more concentrated on the aluminum side, resulting in a shallower pool width at the top and middle of the steel side. In contrast, the aluminum side has the deepest weld penetration.

However, when the weld spacing increased to 2.0 mm, the steel side preheating temperature of the second weld continued to decrease. Additionally, the temperature gradient and Magrani force on the molten pool surface increased, resulting in the laser energy absorbed at the bottom of the keyhole dispersed with the flow of molten liquid on the molten pool surface. Therefore, the pool width of the steel side of the second weld is increased, and the pool width and weld penetration of the aluminum side are decreased.

### 3.4. SEM and EDS Analysis of Weld Cross-Section

**Figure 9:**
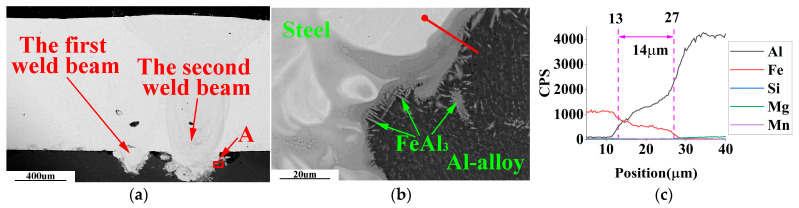
SEM-EDS observation of weldment with weld spacing of 0.5 mm. (**a**) Main areas of weld cross-section; (**b**) enlarged view of area A in Figure 9a; (**c**) EDS line scan results of the red line in Figure 9b.

**Figure 10:**
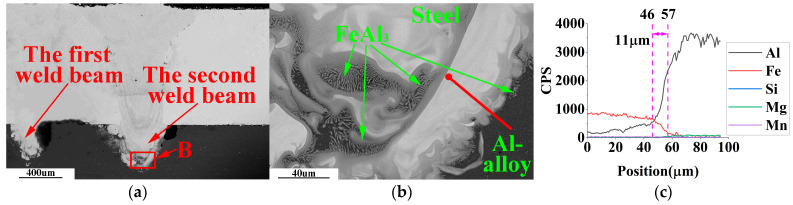
SEM-EDS observation of weldment with weld spacing of 1.0 mm. (**a**) Main areas of weld cross-section; (**b**) enlarged view of area B in Figure 10a; (**c**) EDS line scan results of the red line in Figure 10b.

**Figure 11:**
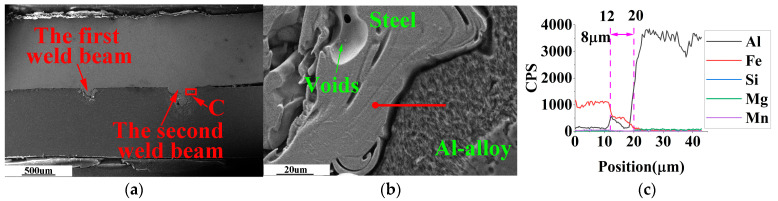
SEM-EDS observation of weldment with weld spacing of 1.5 mm. (**a**) Main areas of weld cross-section; (**b**) enlarged view of area C in Figure 11a; (**c**) EDS line scan results of the red line in Figure 11b.

SEM-EDS analysis of the weldment with weld spacing of 0.5 mm, 1.0 mm, and 1.5 mm in the optimal laser welding parameters, as shown in Figure 9, Figure 10 and Figure 11.

Due to the hexagonal crystal structure of the Fe_2_Al_5_ phase, there are many atomic vacancies on its C-axis. These vacancies are occupied by aluminum atoms, forming needle-like FeAl_3_ phase with disordered growth direction [26]. The needle-like FeAl_3_ is a typical substance in cross-sections of steel-aluminum dissimilar metal welded joints. Thus, the needle-like Fe-Al IMCs in Figure 9 and Figure 10 are FeAl_3_.

In Figure 9b, the white and black parts are the solidification areas of molten steel and aluminum alloy, respectively. On the boundary line between molten steel and molten aluminum, a typical needle-like FeAl_3_ is formed and grows towards the solidification zone of the aluminum side.

Figure 10b shows a black molten aluminum solidification structure inside the white molten steel solidification zone. In laser welding, the metal absorbs heat and evaporates to form recoil pressure on the molten pool’s surface. The molten aluminum enters the molten steel under the stirring action of the recoil pressure. Because the formation and cooling time of the molten pool is short in the laser welding process, the aluminum liquid entering the molten steel cannot fully diffuse in the molten steel, which will form an island aluminum structure in the solidification zone of the molten steel. Many needles like FeAl_3_ grow around the island aluminum structure.

In Figure 11b, no needle-shaped FeAl_3_ was found on the solidification area of steel and aluminum alloy.

In Figure 9c, Figure 10c and Figure 11c, when the welding seams are 0.5 mm, 1.0 mm, and 1.5 mm, the thickness of the steel/aluminum transition layer is about 14 μm, 11 μm, and 8 μm, respectively. Therefore, in laser double-pass reciprocating welding, appropriate weld spacing can change the thickness and type of Fe-Al intermetallic compound in the steel/aluminum transition layer, and improve the tensile strength of the welded joint.

Generally, a higher mechanical strength of weldment has been achieved when the thickness of the brittle IMCs layer has been less than 10 μm [27]. However, the type and morphology of IMCs are highly dependent on the grades of steel and aluminum alloy. Even small changes in melting temperature, molten pool fluidity, solute diffusivity, and thermal conductivity can affect the formation of the IMCs phase [28].

Preheating can adjust the relationship between penetration depth, pool width, and IMCs layer morphology in steel–aluminum dissimilar metal welding.

Ma, Junjie [29] used two laser scans to weld DP590 steel and 6061 aluminum alloy. The results show that the thickness of Fe-Al IMCs can be controlled to about 5 μm under optimized preheating and welding parameters. The thickness of the thinner IMCs layer obtained in our experiment is 8 μm. However, this paper obtained a thinner IMCs layer thickness is 8 μm. The reason for this difference is most likely the model of the laser welding machine.

### 3.5. SEM Observation of the Fractured Surface of the Aluminum Side

In the lap welding of dissimilar metals of steel–aluminum, the fracture surface on the steel side carries part of the solidification zone structure of aluminum, and the fracture surface on the aluminum side depresses downward.

**Figure 12:**
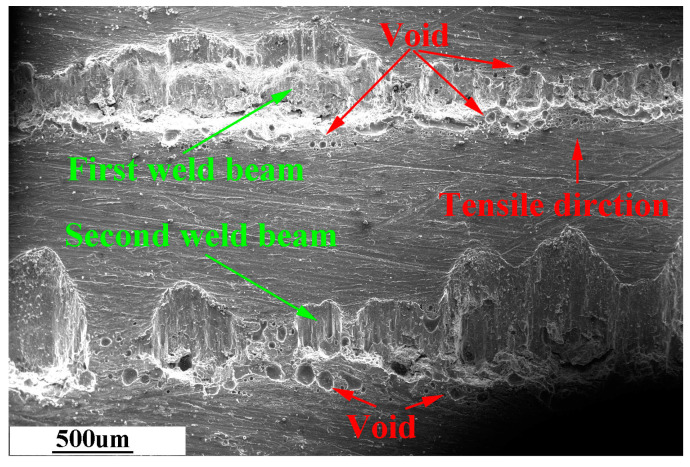
Morphology of the fracture surface on the aluminum side.

**Figure 13:**
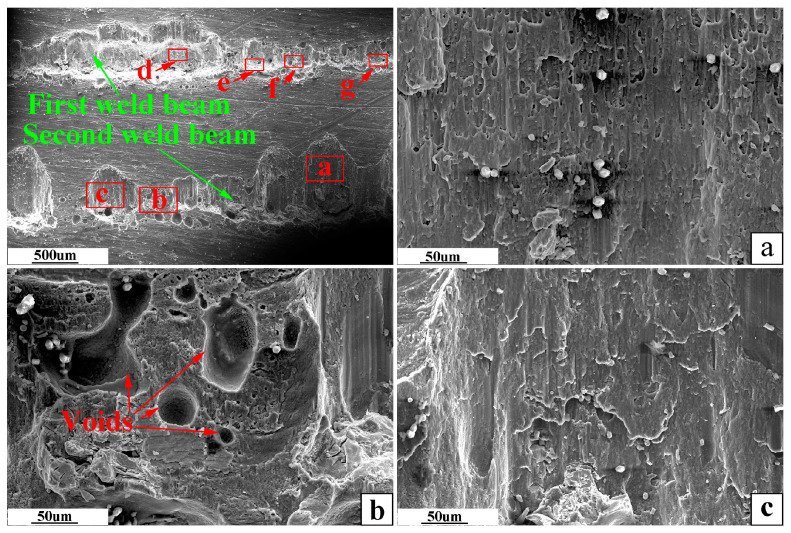
SEM image of the selected area of the fracture surface on the aluminum side. (**a**–**c**) are partially enlarged views of the same red-framed marked area in the upper left picture.

**Figure 14:**
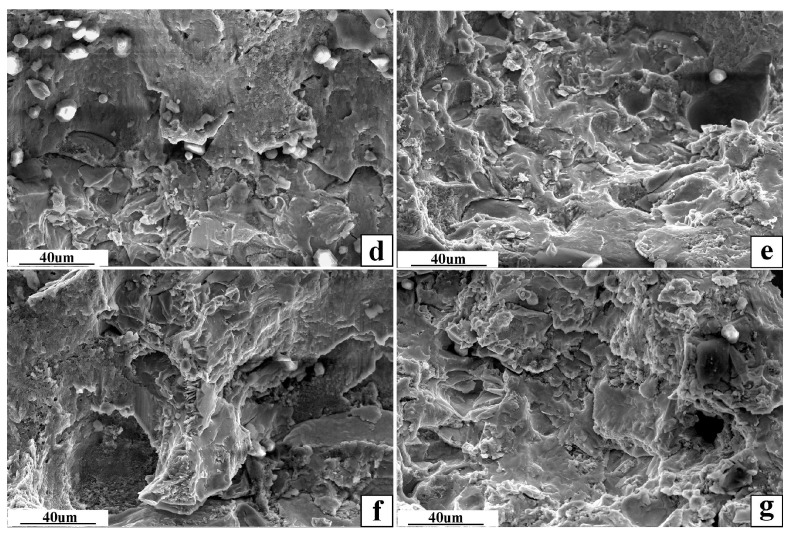
SEM image of the selected area in Figure 13. (**d**–**g**) in Figure 14 are partially enlarged views of the same red-framed marked area in the upper left corner of Figure 13.

The weldment was obtained under the optimal welding parameters and welding spacing of 1.5 mm. SEM observed the fracture surface of the aluminum side of this weldment, as shown in Figure 12, Figure 13 and Figure 14.

In Figure 12, the width of the fracture surface of the first and second weld is not uniform. Moreover, the fracture surface on the aluminum side of the same weld expands along the stretched direction, and the depth of the fracture surface becomes shallower along the stretching force. There are void defects on both sides of the fracture surface of the two welds.

In Figure 13, the first weld fracture surface has an obvious river pattern and cleavage step, which is a typical brittle fracture. The second weld fracture surface has welding defects such as pores. The fracture surface extends to the aluminum base metal, which is mainly ductile fracture.

## 4. Conclusions

This paper studies the influence of weld spacing on the tensile strength, molten pool morphology, temperature history, and fracture surface characteristics of steel-aluminum laser double-pass reciprocating weldment.

(1) With the optimal laser parameters, when the weld spacing is 0–1.5 mm, the welding joint’s tensile strength increases with the weld spacing, and the maximum tensile strength can reach 76.84 MPa. When the weld spacing is 2.0 mm, the weld tensile strength decreases. In double-pass reciprocating welding, the tensile strength of the whole weldment mainly depends on the second weld seam.
(2) Compared with other weld spacing, when the weld spacing is 1.5 mm, the preheating temperature at the steel side of the second weld seam is 421–423 K. The preheating temperature, peak temperature, and pool width on the steel side of the second weld are lower, while the preheating temperature of the aluminum side is 486 K, reaching the maximum. The peak value of weld penetration and molten pool center temperature reaches the maximum on the aluminum side. The thickness of Fe-Al IMCs in the steel-aluminum transition layer of the second weld is reduced to 8 μm. No needle-shaped FeAl_3_ is found near the steel–aluminum transition layer, and the fracture surface of the second weld is brittle fracture features.
(3) The size of the molten pool of steel–aluminum welded joint is affected by preheating temperature, plasma shielding, and smoke shielding.
(4) Preheating welding can adjust the relationship between penetration depth, pool width, and IMCs layer thickness in the laser double-pass reciprocating welding.
(5) Proper weld spacing effectively improves weld forming quality in laser double-pass reciprocating welding of steel-aluminum dissimilar metals.

## Figures and Tables

**Table 1:** Nominal chemical composition of base metals in wt. %.

Materials	Nominal Chemical Composition (wt. %)									
	Mn	Si	Cu	Mg	Cr	C	P	S	Fe	Al
6061-T6	0.067	0.60	0.244	1.098	0.19	-	-	-	0.345	Bal.
DP780	1.99	-	-	-	-	0.094	0.0056	0.0023	Bal.	0.036

## Data Availability

Data is unavailable due to privacy.
